# Cardiac Troponin Elevation and Outcome in Subarachnoid Hemorrhage

**DOI:** 10.7759/cureus.9792

**Published:** 2020-08-16

**Authors:** Tehrim Zahid, Noha Eskander, Mina Emamy, Robert Ryad, Nusrat Jahan

**Affiliations:** 1 Internal Medicine, California Institute of Behavioral Neurosciences and Psychology, Fairfield, USA; 2 Psychiatry, California Institute of Behavioral Neurosciences and Psychology, Fairfield, USA; 3 Department of Research, California Institute of Behavioral Neurosciences and Psychology, Fairfield, USA; 4 Internal Medicine, California Institute of Behavioural Neurosciences and Psychology, Brentwood, USA; 5 Internal Medicine, California Institute of Behavioural Neurosciences and Psychology, Fairfield, USA

**Keywords:** troponin, subarachnoid hemorrhage

## Abstract

Many cardiopulmonary complications occur after aneurysmal subarachnoid hemorrhage. This is due to sympathetic nervous system activation which results in release of norepinephrine from myocardial sympathetic nerves. Cardiac troponin I is a marker for diagnosis of cardiac injury. Elevated levels of troponin in these patients are associated with worse clinical outcomes. PubMed was searched for literature using regular and Medical Subject Heading (MeSH) keywords for data collection. Papers published in English language involving human subjects within the last 20 years focusing on cardiac troponin elevation following subarachnoid hemorrhage were included. Systemic complications that occur after subarachnoid hemorrhage worsen the clinical outcome of patients and have negative effects on the mortality and morbidity of these patients. Cardiac troponin I elevation is significantly associated with the severity of the stroke, poor neurological status, longer ICU stay, and death. Cardiac troponin I should be measured in patients presented with acute stroke. Hemodynamic monitoring and appropriate supportive care can improve clinical outcomes.

## Introduction and background

Subarachnoid hemorrhage (SAH) is followed by systemic complications like cardiac dysfunction which is due to sympathetic nervous system activation and affects the overall outcome of the patients [1,2]. Previously, necrosis of myocardial cells and release of cardiac enzymes were thought to be due to coronary artery disease but now a lot of evidence has proved that it is due to the release of norepinephrine from myocardial sympathetic nerves [3]. Cardiac troponin is a diagnostic biomarker for cardiac damage and it increases in patients of subarachnoid hemorrhage [4]. Troponin levels are considered as normal if less than 0.04ng/ml and high if more than 0.04ng/ml. Troponin levels should be measured after the admission of patients with acute stroke as higher levels are associated with stroke severity and high mortality [5]. There is an increased risk of left ventricular systolic dysfunction, greater neurological damage and a longer stay in the intensive care unit if measured cardiac troponin levels are very high. Perhaps, there is a risk of diastolic dysfunction and pulmonary congestion if cardiac troponin is increased mildly. There is supporting data that subarachnoid hemorrhage has been associated with worse clinical presentation and outcome in patients with an acute cardiac injury which is diagnosed by cardiac troponin I elevation [6]. Cerebral perfusion both focal and global decreases after cardiac dysfunction in patients with aneurysmal subarachnoid hemorrhage. This can be attributed to cardiac dysfunction directly or due to the release of catecholamines in these patients [7]. There is increased intracranial pressure at the time of aneurysm rupture which results in brainstem ischemia, followed by sudden activation in the sympathetic nervous system and increased release of catecholamine [8].

Cardiopulmonary complications occur after aneurysmal subarachnoid hemorrhage which worsens clinical outcome and have a negative impact on morbidity and mortality of patients. The cardiac injury which results in troponin elevation and cardiac arrhythmia are seen in these patients. Therefore, cardiac monitoring and interventions play an important role in patients of aneurysmal subarachnoid hemorrhage as [9]. There are electrocardiographic abnormalities, wall motion abnormalities on echocardiogram and elevation of creatine kinase MB isozyme or cardiac troponin I in patients of subarachnoid hemorrhage. Left ventricular dysfunction in patients with SAH is indicated by sensitive biomarker elevation i.e. cardiac troponin I. This cardiac dysfunction is reversible so during clinical management of these patients hemodynamic monitoring should be strict until the cardiac function returns to normal [10].

This study aims to find the relationship between cardiac enzymes elevation, clinical presentation, and outcome in patients with aneurysmal subarachnoid hemorrhage.

## Review

Method

Data was collected manually on PubMed using Medical Subject Heading (MeSH) keywords. Table 1 shows MeSH keywords for the literature search.

**Table 1:** Medical Subject Heading (MeSH) keywords for the literature search

MeSH KEYWORDS	DATABASE	RESULTS
Troponin subheading aneurysmal subarachnoid hemorrhage	PubMed	112
Subarachnoid hemorrhage subheading troponin	PubMed	76
Subarachnoid hemorrhage subheading cardiac enzymes	PubMed	71

Then studies were selected after applying the following inclusion/exclusion criteria:

Inclusion criteria:

1. Papers published within the last 20 years

2. Human studies

3. Papers published in the English language only

4. All study types

Exclusion criteria:

1. Animal studies

2. Non-English language literature

Results

Table 2 shows the total number of articles selected after applying the inclusion/exclusion criteria in following order using MeSH keywords.

 

**Table 2:** Total number of articles selected after applying the inclusion/exclusion criteria in the following order using Medical Subject Heading (MeSH) keywords.

MeSH keyword subarachnoid hemorrhage (subheading – troponin)	
Total articles	76
Articles selected	66
MeSH keyword subarachnoid hemorrhage (subheading – cardiac Enzymes)	
Total articles	71
Articles selected	22
MeSH keyword troponin (subheading – aneurysmal subarachnoid hemorrhage)	
Total articles	112
Articles selected	100

We obtained 259 articles using the MeSH keywords search. Ultimately, a total of 188 articles were selected regarding inclusion/exclusion criteria as shown in Table 2. Out of the 188 articles, 65 articles were removed as they did not specify the disease of interest (those which did not show the effect of troponin elevation on the outcome of a patient). 

Finally, 35 publications in PubMed were reviewed. Full articles were available for eight publications and 27 publications contained solely abstracts. All records reviewed including eight full articles and 27 abstracts were freely available for review. All studies selected have been in the English language published within the last 20 years.

- Twenty-eight observational studies [2,4-8,10-31].

- Four review articles [1,9,32,33].

- Two case reports [34,35].

- One clinical trial [3].

The maximum number of subjects in the observational study was 1,655 and the minimum was 30. The total number of all the subjects in 27 observational studies were 6140. The clinical trial had 223 subjects.

Discussion

In this review, we analyzed the outcome after cardiac troponin I elevation in the setting of aneurysmal subarachnoid hemorrhage. Our study shows that cardiac troponin elevation after aneurysmal subarachnoid hemorrhage is associated with many systemic complications, poor outcomes and increased mortality.

The present literature review aimed to provide a complete review of the evidence available from various studies to determine cardiac injury evidenced by elevation of cardiac troponin I and sequelae of many cardiopulmonary complications after aneurysmal subarachnoid hemorrhage. It should be noted that many of our studies were analyzed in our literature review. In Table 3, we summarize seven studies. Overall five observational studies and the two review articles explained that cardiac troponin I elevation after subarachnoid hemorrhage is associated with poor outcome and high mortality. However, two other studies summarized in Table 4 showed that cardiac abnormalities are common after aneurysmal SAH but are not associated with increased mortality. The rest of the studies reviewed in the discussion also showed that cardiac troponin is related to poor outcome after subarachnoid hemorrhage.

**Table 3:** Summary of seven studies showing elevated troponin following subarachnoid hemorrhage (SAH) is associated with high mortality.

Author/ Publication year	Study Design	Sample size	Main points	
Ayham M Alkhachroum et al. [5], 2019	Observational Study	1655	TYPE II Myocardial infarction is common in patients with ischemic strokes, intracerebral hemorrhage, and subarachnoid hemorrhage. It is diagnosed by elevated troponin levels. In this study, mortality was 14.7% in ischemic stroke patients, 31.3% in intracerebral hemorrhage patients, and 43.8% in subarachnoid hemorrhage patients with higher troponin levels.	
Maciej Tomasz Wybraniec et al. [33], 2014	Review Article	N/A	After subarachnoid hemorrhage, there is a release of troponin and cardiac dysfunction with ECG changes. Neurocardiogenic injury should be differentiated from acute myocardial infarction. Cardiac dysfunction is associated with increased mortality.	
Nicolas Bruder et al. [9], 2011	Review Article	N/A	In one literature review, the cardiac injury which is diagnosed by an elevation in troponin levels has been noted in about one-third of patients and arrhythmias also occur in about one-third of patients after aneurysmal subarachnoid hemorrhage. Cardiopulmonary complications worsen clinical outcome and negatively affect morbidity and mortality.	
Preeti Ramappa et al. [4], 2008	Observational study	83	Subarachnoid hemorrhage patients with elevated cardiac troponin I levels have worse neurological status at admission in-hospital mortality.	
Rasham Sandhu et al. [14], 2008	Observational study	378	In patients with ischemic stroke, in-hospital mortality is 65% with increased troponin I compared with 4% with normal troponin I. In patients with intracerebral hemorrhage, in-hospital mortality 64% with increased troponin I compared with 28% with normal troponin I. In patients with subarachnoid hemorrhage, in-hospital mortality occurred in 40% with increased troponin I compared 11% with normal troponin I.	
Sirisha Yarlagadda et al. [17], 2006	Observational study	300	Cardiovascular complications after SAH are predictors of patient mortality. In one study, we have 300 patients. BNP level was greater than 600 pg/mL and troponin I was greater than 0.3 mg/L and was associated with death.	
Andrew M Naidech et al. [11], 2005	Observational study	253	Peak troponin levels were linked to increased risk of echocardiographic left ventricular dysfunction, pulmonary edema, hypotension, and delayed cerebral ischemia from vasospasm. Peak troponin levels were predictors of death or disability at discharge but this association was no longer significant at 3 months. Cardiac troponin elevation is associated with an increased risk of cardiopulmonary complications, delayed cerebral ischemia, and death or poor functional outcome at discharge.	

**Table 4:** Summary of tables showing elevated troponin following subarachnoid hemorrhage (SAH) is not associated with mortality.

AUTHOR/ Publication year	Study Design	Sample Size	Main points
Klaudia Urbaniak et al. [16], 2007	Observational study	266	Cardiac abnormalities after SAH do not increase mortality. However, they adversely affect discharge disposition and prolong hospital LOS. In this study, 50% (n = 133) demonstrated cardiac abnormalities as indicated by abnormal EKG, ECHO, or troponin I. There was no difference in mortality between the cardiac abnormality and control groups (P = .33). However, there was increased morbidity in the cardiac abnormality group as demonstrated by worse discharge disposition, in addition to increased length of hospital stay (22.6 vs 17.1 days, P < .01).
Claire E Sommargren et al. [29], 2002	Observational study	100	Electrocardiographic abnormalities like ventricular repolarization has been reported in subarachnoid hemorrhage. In a study of 100 patients with SAH, repolarization abnormalities occurred in 41% of patients, prolonged QTc interval >460 ms in 16%, ST segment elevation in 9%, ST depression in 3%, T wave inversion in 7%, and U wave >or=100 micro V in 15%. Serum cardiac troponin I was elevated in 21%, and was significantly associated with QTc interval >460 ms (P<.001 prolonged="" qtc="" interval="" after="" sah="" is="" related="" to="" myocardial="" injury="" but="" unrelated="" mortality.=""/>

Cardiac Biomarkers

Myocardial necrosis is a neurally mediated process due to the release of norepinephrine from myocardial sympathetic nerves after aneurysmal subarachnoid hemorrhage and results in the release of cardiac enzymes [1]. Cardiac troponin I elevation is used to diagnose neurocardiac injury. In addition to that, Brain natriuretic peptide (BNP) is also secreted in response to injury, inflammation and cardiac myocyte stretch. BNP is significantly associated with cardiac troponin I. For every 1 unit increase in log BNP, troponin increased by 0.05ng/ml (P=.001). For every 1 unit increase in log BNP, patients are 3.16 times more likely to have a poor mRS (modified Rankin Scale) at discharge (P=.021) and 5.40 times more likely at three months (P<.0001). Therefore, both troponin and BNP predict worse outcomes after SAH [2]. Ischemia-modified albumin (IMA), tumor necrosis factor α (TNF-α), and MPO levels were higher by mean values of 11.6%, 9.5%, and 2.9%, respectively, in SAH patients compared with the control group. White blood cells (WBCs), creatine kinase-MB (CK-MB), and troponin values were significantly higher in patients with SAH compared with healthy control (P<.001, P<.01, and P<.05, respectively). WBCs and troponin levels in the circulation correlates with patients’ clinical severity (r=0.598, P=.001 and r=0.461, P=.012, respectively). IMA has poor diagnostic value than creatine kinase-MB and cardiac troponin I in predicting cardiac injury in SAH patients [3].

Pathophysiology

Following SAH, catecholamine-induced neurocardiogenic injury leads to ischemic ECG changes, elevation in cardiac-specific enzymes, neurogenic pulmonary edema, neurogenic stunned myocardium, segmental wall motion abnormalities, stress cardiomyopathy, left ventricular systolic dysfunction and myocardial dysfunction. There are normal myocardial perfusion and abnormal sympathetic innervation in SAH patients with left ventricular (LV) systolic dysfunction. This is due to the excessive release of norepinephrine from myocardial sympathetic nerves, which damage myocytes and nerve terminals [4-7]. Cardiac injury detected by increased troponin after SAH is associated with persistent electrocardiographic and echocardiographic abnormalities like ST-segment elevation, T wave inversion, QTc prolongation, ventricular arrhythmias, regional wall motion abnormalities and decreased ejection fraction [6,8,9].

Cardiac Complications

SAH-induced myocardial dysfunction is associated with high blood pressure, increased heart rate, and ECG changes. Patients with echocardiographic abnormalities like LV diastolic dysfunction and LV systolic dysfunction had two-fold greater plasma BNP, and 40-fold greater troponin. In less severe forms of SAH, there is only left ventricular dysfunction. But in severe forms of SAH, there is excessive release of catecholamines which affect both systolic and diastolic left ventricular function. There is normal atrium volume which suggests an acute alteration in function in isolated left ventricular dysfunction. However, there is global cardiac dysfunction on echocardiography in severe myocardial injury associated with higher levels of troponin I and BNP in severe SAH [10]. Cardiac injury is noted in one-third of patients and arrhythmias in one-third of patients after SAH which negatively affects morbidity and mortality. Cardiac dysfunction defined by myocardial wall motion abnormalities or positive troponin after SAH is also linked to decreased focal or global cerebral perfusion. This relation explains delayed cerebral ischemia after SAH. Therefore, (wall motion abnormalities) WMAs are risk factors for poor clinical outcome after SAH [11,12]. High-grade SAH, elevated troponin, history of prior stimulant drug use and tachycardia are independent predictors of LV regional wall motion abnormalities after SAH. Patients with WMAs have poor Glasgow Coma scale (GCS), poor functional recovery, and increased hospital stay [13-15].

Electrocardiographic (ECG) and Echocardiographic Findings

In another case-control study, peak troponin levels were used to differentiate LV dysfunction (stunned myocardium (SM) associated with SAH) from myocardial infarction in the control group. ECG changes did not match echocardiographic changes in SAH patients. Therefore, troponin values less than 2.8ng/ml in patients with ejection fractions (EFs) less than 40% are consistent with SM and CK-MB trend does not differ between SM and myocardial infarction (MI) but that echocardiograms inconsistent with EKGs indicate SM [16]. Diastolic dysfunction also explains the development of pulmonary edema in SAH patients. This is also linked to the history of hypertension and older age [17].

Systemic Complications

Cardiac troponin I elevation is also associated with pulmonary congestion, acute lung injury, cardiovascular morbidity, delayed cerebral ischemia (DCI), longer ICU stay, poor functional outcome at discharge, and mortality [18-20]. Neurogenic stunned myocardium (NSM) which is the triad of transient LV dysfunction, ECG changes, and elevated troponin resembles myocardial infarction but has good prognosis if managed early and appropriately [21]. Aggressive hemodynamic monitoring should be done until cardiac functions return to normal [22]. Serial troponin measurement, continuous ECG surveillance, for patients with poor clinical presentation and neurological examination can result in early detection of SAH induced myocardial infarction which will alert staff to observe patients for clinical manifestations of subarachnoid hemorrhage-induced myocardial infarction (SAHMI) and provide supportive care and treatment which in turn improve clinical outcome and reduced mortality.

Further studies are warranted to explore more consequences of troponin elevation, early detection methods, monitoring protocols, and treatment strategies to decrease mortality and morbidity.

Limitations

The current literature review has some limitations. It includes studies conducted within the last twenty-year period. This results in variations in the care of patients and changes in assays of cardiac biomarkers. There was no age-specific or gender-specific analysis performed. There are many unexplored factors that can be tested in further studies.

## Conclusions

After analysis of data, we found out that myocardial injury indicated by an elevation in cardiac troponin I occurs after aneurysmal subarachnoid hemorrhage. This is due to sympathetic nervous system activation and release of norepinephrine from myocardial sympathetic nerves after aneurysmal subarachnoid hemorrhage rather than coronary artery disease which was once thought to be the cause. Many systemic complications occur after subarachnoid hemorrhage like myocardial dysfunction, neurogenic pulmonary edema, neurogenic stunned myocardium, segmental wall motion abnormalities, stress cardiomyopathy, and delayed cerebral ischemia. These cardiopulmonary complications have been linked to worsened clinical outcomes in these patients and negatively affect morbidity and mortality. Cardiac troponin I elevation directly correlates with the severity of a stroke, poor neurological status, longer ICU stay, and death. It should be considered in patients who present with acute stroke. Hemodynamic monitoring and appropriate supportive care can improve clinical outcomes. Further research is needed to investigate and identify more quick and effective ways to monitor patients for neurocardiogenic injury in the acute phase of hemorrhage.
